# Floating at COP30: exclusivity and the carbon cost of travel and accommodation in Belém

**DOI:** 10.14324/111.444/ucloe.3595

**Published:** 2026-05-28

**Authors:** Joffrey Doma, Simon Chin-Yee, Priti Parikh, Jonathan Barnsley

**Affiliations:** 1 Department of Political Science and School of Public Policy, University College London, UK; 2 Bartlett School of Sustainable Construction, University College London, UK; 3 Department of Geography, University College London, UK

**Keywords:** COP30, emissions, carbon footprint, cruise ships, accommodation, Belém, conference, transport, travel, climate change

## Abstract

Conference of the Parties 30, the 2025 annual United Nations Framework Convention on Climate Change conference, took place in Belém, Brazil, located right on the edge of the Amazon forest. It was championed by Brazilian authorities as a symbolic centring of the Global South. Nonetheless, as previous studies in this series have shown, long-haul air travel often dominates total emissions, and this was the case for COP30. However, Belém presented a new logistical challenge. Both its location and general lack of adequate accommodation prompted the authorities to charter two large cruise ships to house thousands of delegates over the two weeks of the conference. The expected operational emission from such ships makes them several times more carbon-intensive than standard hotels. Yet the challenge was compounded by the affordability problem for delegates from the Global South, while the complete lack of affordable and accessible accommodation for civil society organisations directly contradicted the symbolic inclusion echoed by the organisers. This paper reports on travel emissions to Belém, highlights the paradox of housing delegates on carbon-intensive cruise ships, as well as the contradictions of not catering to civil society organisations in a supposedly inclusive COP. This paper concludes with a recommendation that subsequent conferences should endeavour to also account for accommodation emissions from participants.

## Introduction

The United Nations Framework Convention on Climate Change (UNFCCC) held its annual Conference of the Parties (COP30) in Belém, Brazil, from 10 to 21 November 2025. The choice of location was strikingly symbolic. Belém is located at the gateway to the Amazon and hence presented an opportunity to highlight the struggles of indigenous communities, ecological fragility and the critical role of tropical forests in climate governance. A recent synthesis work by Flores et al. [1] warns that the Amazonian forest ecosystem presently faces compounding issues – from warming to droughts and deforestation to fires – with 10–47% of forests now potentially exposed to conditions that could precipitate abrupt ecosystem transitions by 2050. Nonetheless, symbolic gestures could not obfuscate the complex and significant logistical and environmental difficulties associated with holding a conference of that magnitude in a relatively small and inadequately connected city. Infrastructure projects were implemented to improve access to the COP30 venue, but that in itself had a detrimental environmental impact, especially on the rainforest and local communities [2]. In the past, arguments about COPs being held in cities without the infrastructure to host the numbers of people attending have been addressed – at COP24 Katowice, there were complaints from many delegates that some would have to commute from Warsaw because the city was too small, or that at COP27 Sharm El-Sheikh, the prices for hotels and resorts were so prohibitive that many had to travel from as far as Dahab (2 hours away). Scholars have noted the institutionalisation of climate summits, which has effectively transformed COPs into complex mega-events, and as such brings to the fore concerns about participation, legitimacy and the environmental impact of hosting such global negotiations [3,4]. Still, some studies have critiqued the performative elements of climate conferences, suggesting that such occasions tend to simultaneously serve as negotiation forums, a political spectacle and a symbolic signalling (as in the case of Belém) within global climate politics [5].

In the case of Belém, controversies had emerged even before the conference commenced. Simply reaching Belém was always going to be challenging for delegates, civil society and the media. Unlike previous COP locations in Europe or the Middle East, there are no straightforward or direct road or rail connections from major international gateways. Unfortunately, long-haul flights were unavoidable, with the standard routes from Europe and North America requiring at least one or two connections in cities such as Lisbon, Miami, São Paulo and Rio de Janeiro. Most participants required further domestic flights once in Brazil – which added to both the costs and increased greenhouse gas (GHG) emissions. Previous studies show that travel to conferences represents the largest source of carbon footprint, dwarfing emissions from location operations or catering emissions [6,7]. Belém was not an exception. Participants from many Small Island Developing States (SIDS), especially from the Pacific, would need to take multiple flights to reach Belém. Some organisations had to cut back on participation due to the logistical challenges, which had a knock-on effect in terms of representation at the conference. Hence, the contribution of this analysis is not in highlighting previously unknown sources of emissions, but rather in putting into context the logistical arrangements of COP30 – especially in the deployment of cruise ships for accommodation – as a useful empirical illustration of broader tensions between environmental goals, accessibility and infrastructural constrictions in global climate governance.

A lack of adequate housing capacity inspired the Brazilian authorities to charter two large cruise ships to provide temporary accommodation for government delegates [8]. As highlighted in the Climate Home News report, this was considered by organisers as a pragmatic response; however, the solution sparked controversy. Cruise ships are among some of the most carbon-intensive means of transport and accommodation: they consume large quantities of energy for air conditioning, lighting, catering and entertainment [9]. This has led to cruise ships being dubbed as ‘floating cities’ or ‘floating hotels’ [10,11], with daily emissions per passenger on cruise ships exceeding those of aviation. Interestingly, the timing of the conference meant that it commenced in the immediate aftermath of the International Maritime Organisation’s (IMO) refusal to adopt what should have been a breakthrough Net Zero Framework (NZF) on global shipping, which aimed to combine compulsory emission limits with GHG pricing across the maritime industry [12]. It is curious that while this agreement is generally considered a breakthrough, many Latin American countries (Argentina, Chile, etc.) were staunchly opposed to it, arguing that further costs on maritime trading could hamper their development goals [13]. It was in fact Brazil that ended up pushing for the approval of this framework in the Maritime Environmental Pollution Committee (MEPC) meeting in October 2025. Nevertheless, the actual NZF that the IMO member states voted on was significantly watered down by Brazil and other emerging countries from what was seen as a more ambitious framework with a fuel standard without flexibility, not to mention a GHG levy of $150 per tonne of carbon dioxide equivalent (CO_2_ eq), which would have provided greater certainty of hitting the 2050 net zero targets [14]. This tension underscores the intersecting issues of climate ambition, equity and national interest. Moreover, the decision to reserve cruise ship accommodation strictly for government delegates highlighted questions of equity and access, with the conference potentially sidelining civil society organisations.

This paper is a critical analysis forming part of a series of papers that examine the carbon footprint of partaking in UNFCCC COPs. The paper combines two analytical approaches: quantified estimates of emissions from travel from the UK to Belém using the University College London (UCL) carbon footprint calculator, and literature-based modelling analysis of potential emissions from cruise ship accommodation during COP30. Following earlier analyses of travel emissions by delegates to past COPs [6,7,15], this paper reflects on the logistical and environmental implications of COP30 in Belém, relying on estimated emissions from an established travel carbon calculator. The paper, therefore, aims to analyse travel and accommodation emissions associated with participation in COP30 from a UK departure point, and situate these estimates within a broader conversation on the governance, logistical and equity implications of hosting large-scale climate negotiations in geographically remote locations. A growing body of scholarship has questioned the scale and governance of contemporary climate summits, highlighting that COP negotiations have expanded into ‘mega-events’ involving tens of thousands of participants beyond the formal negotiators [16]. As Falkner [17] notes, the rapid growth of COPs raises concerns associated with logistical feasibility, participation equity and the environmental impacts of travelling to these negotiations. Unlike recent works in the series that have paid attention to the carbon costs of international travel to COP – such as carbon emissions from private jet journeys [15] – this paper particularly centres on the potential carbon costs of accommodation as well. It is worth highlighting that the emissions calculator utilised here is not a novel methodological contribution, having been innovated in Barnsley et al. [7], but an applied analytical tool deployed to estimate travel-related carbon emissions in a transparent and comparable manner. The use of cruise ships to accommodate delegates in Belém is considered a significant and controversial contributor to COP30’s carbon footprint. Accounting for this is crucial not merely for accuracy but also for transparency, especially if we are to ensure that such aspects of conference organisation are not obscured in future carbon reporting. The rest of the article outlines the methodology behind the updated carbon calculator for COP30 and goes on to discuss the resulting approximate emissions from travel and accommodation, their political and equity implications, and concludes with recommendations for the cruise ship industry and future COPs.

## Methodology: the carbon footprint calculator

While the carbon footprint calculator discussed here (available from UCL Climate Hub) produces actual quantitative estimates for flights from the UK to Belém, a similar methodological explication is not provided for the cruise ships and accommodation generally, because these rely primarily on scenario-based modelling utilising emission factors reported in existing shipping and cruise ship industry studies and reports (see Appendix for further information on data sources, assumptions for cruise ship modelling, and formulas for the carbon calculator). Updating the UCL carbon footprint calculator for COP30 in Belém follows three previous versions: the first for COP27 [7], the second for COP28 [15] and the last one for COP29 [6]. This version uses the same core framework as earlier iterations: it is modular with four transport components (aircraft, rail, car, coach), connected by networks of origin-destination points. In the first paper in this series, Barnsley et al. [7] provide a detailed explanation of the calculator’s framework. In line with the Intergovernmental Panel on Climate Change/European Environment Agency (IPCC/EEA) guidelines, the calculator utilises a ‘tier’ approach: the flight component employs a high-detail tier 3 energy/burn model, while road and coach employs simpler top-down factors (tier 1) and rail utilises a semi-aggregated country-level method (tier 2) (also see IPCC [18]; EEA [19]).

As detailed in the first series, distinct pathways calculate emissions for flight segments, road and rail based on distances and vehicle/energy data. The module for road/coach utilises user input and the type of vehicle (fuel or electric) and using reported standard fuel-efficiency information calculates CO_2_e/km. For rail, the calculator divides every journey into country segments, converting passenger-km into CO_2_e based on national electricity-emission factors. And the flights module divides flights by landing–take-off (LTO) and cruise phases; the data for this are from published per-aircraft LTO fuel burns and the Breguet range equation for cruise fuel, from which the calculator then converts fuel to CO_2_e based on the global warming factors outlined in the IPCC’s sixth Assessment Report (AR6) [20]. The sum total of the modules outline the total footprint for each route.

Routes are designed by connecting major cities: the default origin on the calculator is London (it could be any one of seven other UK cities), and the calculator identifies feasible multi-stop routes to the conference location. Flights were identified utilising flight schedule data (from FlightConnections and Google Flights), with journey dates typically selected for around two days before the conference began on the 10 November 2025. The road/coach/rail segment (primarily for within Europe journeys) are drawn from Google Maps API, while flight distances have been calculated using the great circle routes (orthodromic) formula. As in previous versions, any routes that cross ‘no-travel’ regions have been omitted.

For each candidate’s flight leg, the fuel consumption has been calculated bottom-up. Aircraft type and seating data have been sourced from publicly available sources and flight schedules (mainly Flight Connections and Google Flights). The LTO fuel burn data are based on EEA average figures per aircraft model, and cruise fuel is computed using the Breguet range equation. Total fuel (LTO + Cruise) is converted into CO_2_e by multiplying by 3.15 (IPCC CO_2_ factor) and by 1.9 to report for non-CO_2_ warming effects. This calculator maintains Barnsley et al.’s [7] 1.9× radiative forcing multiplier based on standard IPCC/ICAO practice [18,21]. The outcome is a CO_2_e-per passenger value; premium-class seats are assigned a ‘seat multiplier’ as accounted for in Barnsley et al. [7].

As noted, the rail component is based on a tier 2 country average methodology and is primarily for networks within Europe. The method combines each country’s total rail passenger-km and rail electricity usage (2019 data) to arrive at a kg CO_2_e per passenger-km (albeit implicitly, this averages out over electric and diesel trains). The footprint of the country traversed is assigned for every segment of a journey; and the network mix is approximated by scaling passenger-km by 0.8 (which reflects the electrification of 80% of European Union (EU) passenger traffic [7]). This is to say that a journey through France uses a low-carbon grid factor, while one through Bulgaria, for instance, will use a higher factor.

For cars – using a tier 1, per-km factor – fuel economy data is sourced from EU Worldwide Harmonised Light Vehicles Test Procedure (WLTP) figures and standard emission factors are then applied for petrol/diesel (kg CO_2_/litre) or electricity. For channel crossings, the Eurotunnel use (2 kg CO_2_e/car) is assumed, with expected minimal impacts on long journeys. For coaches, emissions are computed by multiplying the same road distance by a fixed per-km diesel coach factor from the UK’s emissions database. Because full occupancy is assumed for coaches, the per-passenger footprint is expected to be relatively low. It is worth highlighting, however, that while overland travel is practical within Europe (e.g., London–Lisbon by rail), such journeys have been excluded from the primary result analysis because, at the end of the day the long-haul transatlantic flight segment bears a much greater burden in terms of total emissions. Hence, excluding overland short leg journeys within Europe does not in reality have any significant bearing on the overall findings given that getting to Belém requires a transatlantic flight and most likely one or more domestic flights once in Brazil. Indeed, previous analysis in this series confirms this: intra-European ground legs contribute marginally (<10%) to the overall emissions once we account for transatlantic flights [6,7].

All in all, the updated calculator closely follows the original and established design. All distance data, data on vehicle efficiency and emission factors are drawn from publicly available sources (Google APIs, IPCC standards, Biodiversity Information System for Europe/EEA databases), and users are able to see total CO_2_e as well as estimated travel duration for each route. As noted earlier, the methodology is primarily adapted from Barnsley et al. [7], where major details are thoroughly discussed. This paper tries to maintain the ethic of transparency and reproducibility introduced in Barnsley et al. [7] and later in Roberts et al. [15], and more recently in Brown et al. [6].

## Results: travel emissions

From a thorough engagement with flight schedule information available on FlightConnections and Google Flights, there are no direct flights available between the UK and Belém; each possible route includes at least one stop (e.g., via Lisbon, Rio de Janeiro or Bogotá). Drawing data from Google Flights and FlightConnections we are able to construct representative itineraries and calculate great circle distances and flight times (see Table 1).

**Table 1.** Summary of representative air routes London–Belém (using data from FlightConnections and Google Flight)

Route (via)	Distance (km)	Time (h)	CO_2_e (kg)
London–Lisbon–Belém	7565	11.0	~908
London–Rio–Belém	11,697	15.1	~1170
London–São Paulo–Belém	11,914	15.4	~1191
London–Bogotá–Belém	11,397	15.5	~1140
London–Miami–Belém	11,664	15.7	~1167

**Figure 1:**
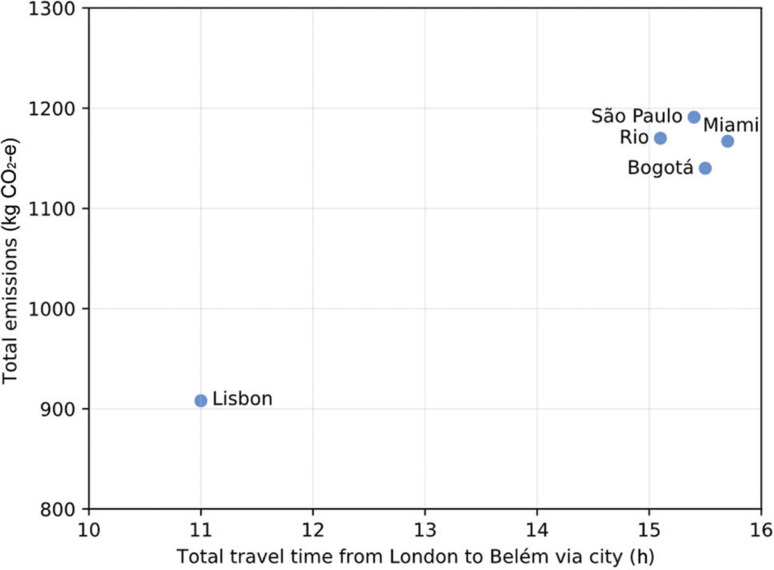
Scatterplot of travel time against GHG emissions for flights from London to Belém as in Figure 3.

There are obvious carbon trade-offs between distances and stops. The connection via Lisbon has the shortest duration (11 h vs. 15–16 h for others), with emissions of 20–25% less per passenger. Other longer routes generate significantly more emissions (Fig. 1). For example, even the most favourable non-European route (via Bogotá) incurs emissions of around 1.14 t compared to 0.91 t for Lisbon. As evident from previous iterations of the calculator in this series, the longest times naturally correspond with more carbon-intensive journeys. The São Paulo route, for example, adds 4.4 h and 300 kg CO_2_e more than the Lisbon route, generating 1.19 t. Contrarily, in principle at least, marginal increases in time could reduce emissions; however, there are no practical longer-land routes for delegates to Belém. Every viable option for delegates from the UK will require a transatlantic flight, therefore it is impossible to attain any dramatic emissions savings similar to UK–Europe overland journeys for COPs (see [6,7]).

**Figure 2:**
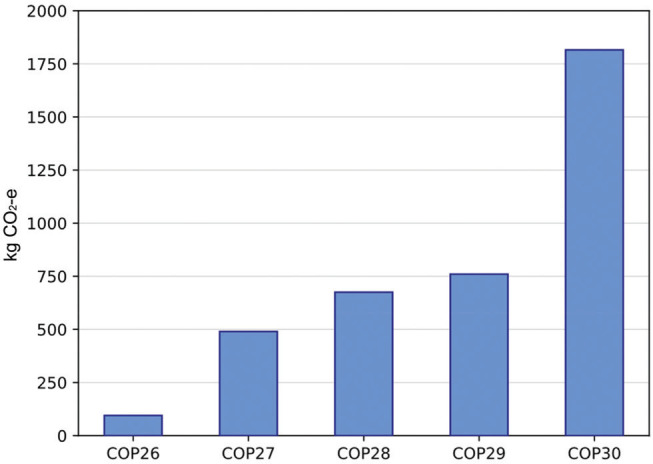
Emissions associated with direct flights from London to recent COPs compared against this year’s most carbon-efficient route to Belém via Lisbon.

Overall, travelling to Belém incurred a much larger carbon footprint for UK delegates, than previous COPs (Fig. 2). For context, a direct flight (4000 km) from London to Baku generated 0.76 t CO_2_e [6]. Even while excluding airspace detours, London–Belém is almost double the distance and approximately double the emissions. However, there are marginal offset gains, which involves the use of larger, long-haul jets (LATAM 777/787 or TAP A330) that burn between 0.10 and 0.12 kg CO_2_e per passenger-km, instead of the older A321 that might have been used for travel to Baku (with around 0.19 kg/km). Nonetheless, the pattern holds: the longer the routes (and more layovers), the higher the carbon emissions per passenger. The utter scale of travel to Belém (≥1.1 t vs. 0.76 t to Baku) underlines the ways conference venues can drive inequality in travel emissions.

In line with previous iterations of the carbon footprint calculator, an underlying assumption for predicting flight itineraries is that a majority of the delegates would have commenced their journeys one or two days before the official first day of COP (8 and 9 November 2025 for Belém). Even after a successful flight into one of the connecting cities in Brazil, it was almost impractical to complete the journey to Belém by land. A major factor driving this is that the inter-city rail network is non-existent in Brazil. There are only two passenger lines (the Carajás mineral railway in Pará and another in Minas Gerais); neither of these offered any feasible connection to Belém. The latest statistical report from Brazil’s national agency for transportation – Agência Nacional de Transportes Terrestres (ANTT) – shows that over 90% of rail use in the country is primarily for freight purposes [22]. Another factor is that while travel by coach is a possibility, it would have been extremely slow at getting delegates to the conference on time based on the assumed one/two days arrival period before the commencement of the conference. For instance, a single journey by bus from Rio de Janeiro to Belém is about 2450 km (around 2 days and 6 hours or 54 hours in total), it is even longer for São Paulo–Belém (3300 km). Data from Brazil’s own largest domestic coach network – ClickBus as well as Busbud (a global booking and data platform) – provide estimates of travel times and distances by bus for verified schedules within Brazil. Durations such as these exceed the 6–8 hours domestic flight times by some distance (such as Rio to Belém = 3.5 h, or São Paulo–Belém = 3.7 h).

However, it is worth noting that the Brazilian authorities constructed a four-lane highway through the Amazon forest, in hope of facilitating easier transport to and from the conference venue in Belém [23]. Aside from the obvious controversy of the destruction of the Amazon, the carbon implications for travel to the conference via the roads had minimal if any effects on overall emissions. Nonetheless, delegates did not have any credible alternatives to flying once in Brazil, as hypothetical rail transfers or coach journeys would have significantly increased travel time without any simultaneous real carbon benefits (with most coach routes requiring at least one overnight stay).

**Figure 3:**
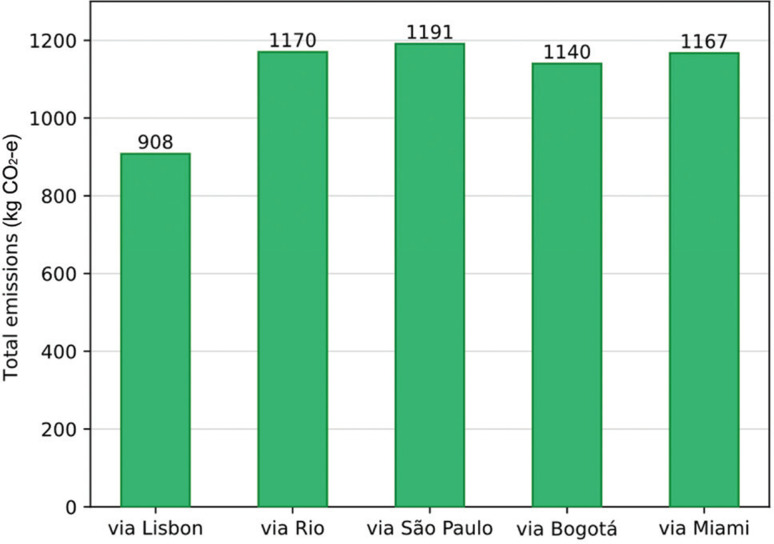
Calculated GHG emissions for flights from London to Belém with a stop at one of five candidate cities.

In summary, the updated UCL travel carbon footprint calculator indicates a wide variation in one-way emissions to COP30 in Belém (estimated around 0.9–1.2 t CO_2_e per passenger from London). As Fig. 3 shows, the shortest connection within Europe (via Lisbon) offers the lowest travel emissions (1 t CO_2_e), while other longer routes incur a carbon cost of 1.2 t CO_2_e. The range (±25%) in emissions underlines a trade-off: delegates would have been able to save 300 kg CO_2_e by travelling for four more hours (London→Lisbon→Belém instead of London→Brazil→Belém). Broadly, these results highlight the accessibility – inequality tension at COP30. Travelling from other parts of the world such as Africa and the Pacific to Brazil was expected to prove to be even more arduous, more carbon-intensive, expensive and possibly off-putting for many from civil society organisations. Data from the Brazilian Institute of Geography and Statistics (IBGE) partially confirm this challenge, with Belém’s regional inflation explosion partly driven by a +25.32% increase in airfares during the COP window [24]. As previously discussed by Brown et al. [6], host locations for COPs tend to privilege delegates in close geographical proximity; in this case the lowest-emitting route (via Lisbon) would still have 50% higher emissions than getting to Baku from London. While travellers from the UK could prioritise the Lisbon route (or potentially split journeys via multiple EU hubs) and maximise occupancy (i.e., full flights), it would have also been prudent for the organisers of COP30 to transparently consider the carbon implications of the origins of attendees.

### Analysis: modelling cruise ships emissions

The issue of emissions from accommodation up until now has been peripheral to the overall conversations on the carbon cost of hosting COPs, but the decision of the Brazilian authorities to accommodate delegates aboard two cruise ships significantly altered that position. The following discussion is a scenario-based analysis of potential estimates of cruise ship accommodation emissions during COP30; it relies on values reported in the literature rather than using vessel-specific operational primary data. The estimates are derived from published ranges of cruise ship fuel consumption and auxiliary engine loads reported in the literature, and hence only reflects indicative scenarios rather than precise measurements for the *Costa Diadema* and *MSC Seaview* (the ships used in Belém). The analysis treats these vessels primarily in their docked mode as stationary accommodation platforms in Belém. Hence, estimates on emissions only incorporates auxiliary engine power generation (‘hoteling’) associated with onboard services (air conditioning, lighting, catering, etc.), while deliberately omitting propulsion-related emissions from sailing or repositioning of voyages. Distinguishing between ‘sailing’ and ‘hoteling’ emissions is a useful analytical boundary for evaluating the implications of using cruise ships as temporary accommodation infrastructure in Belém. This is consistent with extant literature that separates emissions associated with sailing during voyages from auxiliary power demand while ships are at berth [25,26].

Cruise ships today are described as self-contained, floating cities, which burn vast amounts of fuel to propel themselves and provide power for services on board similar to those in hotels [27]. Moreover, other modelling studies suggest that hotel-like electricity loads can account for close to 20% of total energy use in a ship’s hotel system [27]. Public sources indicate that large cruise liners have the ability to consume close to 250 tons of marine fuel per day on a regular basis [28,29], emitting an approximated 750–800 kg CO_2_e per ton of heavy fuel oil—comparable to around 200 tons of CO_2_e per day.

**Figure 4:**
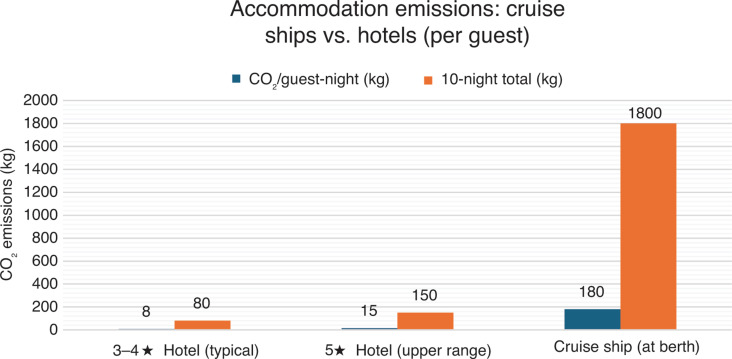
An estimate of accommodation emissions per guests-night: hotels vs. cruise ships at berth (in-port auxiliary power). Hotel factors (typical 3–4 star = 6–10 kg; 5 star = 12–18 kg CO_2_/guest-night) and cruise hotel-load synthesis giving 150–200 kg CO_2_/guest-night for large ships without access to an onshore power supply.

The vast quantity of carbon emitted from cruise ships originates from operational fuel use. Research indicates that small cruise vessels emit an estimated 85 t CO_2_ daily with 27 t of fuel, while larger ships emit between 400 and 800 t CO_2_ burning between 140 and 250 t of fuel per day [28,30]. The International Council on Clean Transportation using verified data from the Royal Caribbean’s cruise liner *Anthem of the Seas* found emissions of 317 g CO_2_ per passenger-nautical mile, equivalent to 0.75 t CO_2_ per-passenger weekly [25]. This data indicates that a passenger on a cruise ship produces about eight times more carbon footprint than standard air or land-based holidays [9]. As Fig. 4 shows, publicly available data indicates that passengers onboard large cruise liners generate around 15–20 times more emissions per night compared to hotel stays. This illustrates the substantial disparity in the intensity of emissions between conventional hotels and cruise ships used as floating accommodation. Standard (land-based) hotel stays are – based on assumed data derived from official information such as the UK Department for Energy Security and Net Zero (DESNZ) hotel factors [31] – estimated to generate between 80 and 150 kg CO_2_ per-guest across a 10-night conference stay; while cruise ships operating without shore power may generate between 1.5 and 2 tonnes of CO_2_ per-guest over the same period. This is reflective of the high energy demand of onboard hotel services.

Furthermore, beyond CO_2_, cruise ships also produce methane [particularly from liquefied natural gas (LNG) engines] and black carbon, which are both known powerful climate forcers [32]. It is estimated that in 2022 cruise traffic in Europe produced 8.1 Mt CO_2_, with methane slip having risen five times to 7800 t and black carbon adding a further 10% to cumulative warming potential [32]. As highlighted by the Cruise Lines International Association (CLIA), LNG is useful for cutting sulphur and nitrogen emissions, but spillage from its methane can offset such gains unless engines are capable of attaining 1% slip [33].

Additionally, as already noted, the focus in this study is exclusively on emissions from in-port hoteling factors. While the highest percentage of emissions from ships are generated during sailing, propulsion-associated fuel usage falls outside the scope of this analysis because the focus is primarily on the accommodation infrastructure of the ships while docked and the implications of its usage at an international climate conference. Indeed, while these particular emissions may be smaller, they remain analytically critical given that the per-guest emissions associated with cruise ship accommodation are substantially higher than conventional land-based hotels. Similarly, it is worth highlighting that while aviation explicitly dominates overall emissions associated with participation at COPs, the focus on cruise ships remains analytically relevant because it highlights the carbon implications of the particular accommodation strategy deployed at COP30. Therefore, this analysis does not suggest that cruise ship emissions dominate, rather it illustrates the added emissions associated with this specific logistical solution.

In any case, absent of any onshore power supply (OPS), the auxiliary engines from docked cruise ships at port continue to burn marine fuel for air conditioning, lighting and desalination (hotel loads) – estimated at around 2–10 MW – producing about 6 t CO_2_ hourly for a 10 MW load [34,35]. Only a marginal number of global cruise ports presently provide shore power [36]; however, both Norway’s ‘zero-emission fjords’ mandate (2026) and the EU’s FuelEU Maritime Regulation are aiming to extend this coverage [36,37].

It is requisite to briefly and explicitly note the useful distinction between operational and embodied emissions. As already discussed above, operational emissions (burning fuel during service) account for the vast amount of a cruise vessel’s total carbon footprint. Embodied emissions, however, which refer to carbon footprint that pertain to lifecycles from construction, maintenance and dismantling, primarily produces emissions from high-carbon materials such as aluminium, glass and steel [38]. Other contributing factors include port dredging, terminal construction and shipyard activities [39,40]; while downstream influences such as food provisioning, on-shore excursions and waste processing extend footprint production beyond vessels [41,42]. Even before setting sail, the infrastructure that underpins cruise tourism carries a significant embedded energy and material cost.

## Assumptions: cruise ships hoteling emissions in Belém

### The *MSC Seaview* and *Costa Diadema*

In an effort to provide adequate accommodation for delegates during COP30 in Belém, Brazilian authorities chartered two cruise ships: the *MSC Seaview* and the *Costa Diadema*. The cruise ships were expected to increase accommodation capacity in Belém and provide around 6000 beds for international delegates. Hotels in Belém are only able to offer around an estimated 18,000 beds, while over 50,000 participants were initially expected at COP30. The cruise ships are treated as stationary accommodation rather than transport vessels. Hence, the analysis focuses on hoteling emissions, defined as energy generated by onboard auxiliary engines to power the ships for hotel services such as air conditioning, lighting, food preparation and water treatment. This analysis excludes the emissions that would have been generated from sailing the vessels to and from Belém because the study’s primary focus is on the incremental emissions generated by the use of cruise ships as stationary accommodation during COP30. This analytical boundary isolates emissions associated with the official two weeks of the conference itself, rather than try to account for the full operational lifecycle of the vessels. Given that the analysis uses assumptions derived from published ranges of cruise ship hotel-load energy demand reported in the literature, adding sailing emissions would introduce significant uncertainty as these voyages are dependent on vessel deployment decisions and itineraries that cannot directly be attributed to the conference.

Both ships are large ocean-going cruise liners—approximately 130,000–154,000 GT and offer a combined guest capacity of between 6000 and 7000. Normal operations for ships of this size are estimated to consume 200–250 t of fuel daily and produce around 150–200 t CO_2_ per day [30]. For comparison, Fig. 4 illustrates the remarkable difference in emissions generated per person for cruise ship lodgings and standard hotel stays. According to CLIA, however, 52% of ships (61% of global capacity) are now OPS-capable, but very few ports in Brazil possess this capability and Belém is not one of them [36]. Consequently, the lack of OPS infrastructure at the newly built cruise terminal at Porto do Outeiro specifically for COP30, indicates that the *MSC Seaview* and the *Costa Diadema* relied on onboard generators during the conference, potentially resulting in the emission of several dozen tons of CO_2_ daily. As reported by Climate Home News [8], this was confirmed months before the conference via email from a spokesperson for the Brazilian authorities that the ships would rely on onboard generators to power air conditioners, televisions and other equipment. The same official communication explained that the generators would utilise a variety of fuel sources, from conventional diesel to biodiesel.

While the two cruise ships provided an innovative solution to Belém’s housing shortage, they also likely added significant emissions to the conference’s overall footprint, which can be compared to thousands of car trips.

## Limitations: current decarbonisation measures

While progress has been made in cutting the carbon footprint of the cruise industry, this remains limited. On the one hand, as highlighted by CLIA [36], more than half of cruise ships (61% global capacity) are now OPS-capable, which allows ship engines to be shutdown at port and potentially cut emissions by around 98% depending on the electricity mix. On the other hand, however, only around 3% of global cruise ports presently have the right infrastructure to deliver on this. Investment in green port infrastructure is in its infancy, and it may take years to adequately accommodate the cruise industry; the recently pushed back IMO NZF would have provided some certainty of how and when this industry will need to move forward in decarbonising. Regardless, it was never going to be on time for Belém.

Efforts to decarbonise propulsion has around 7% of cruise ships operating on LNG, cutting down on local pollutants, but mitigated by marginal carbon benefits from methane slip [32]. There is a growing move towards the fitting of vessels to be able to use methanol and biofuels, and about 15% of ships now integrate fuel cells or battery-hybrid systems, allowing for short intervals of zero-emission operation [36]. Moves are also being made to deploy incremental energy-efficient technologies such as waste-heat recovery, smart heating, ventilation and air conditioning (HVAC) and lighting management, and air lubrication systems [30]. Nonetheless, at present, even where these advances have been deployed, overall emissions reductions are meagre. Indeed, one cruise ship generally produces more carbon per passenger than most forms of leisure travel [32]. Implementing such measures would not have yielded any significant cuts in emissions at the Belém port for COP30.

## Discussion: implications of cruise ships for accommodation at COP30

While travel and especially long-haul flights clearly dominate emissions associated with delegates travel to COP, the use of cruise ships as temporary accommodation highlights further and separate questions about the carbon implications of conference logistics. In that regard, COP30 was particularly unique because it brought to the fore not just the carbon costs of hosting and travelling to climate summits, but also because of the wider debates that material and logistical constraints and solutions sparked on accessibility, exclusivity and equity in global climate governance. Often referred to as the ‘gateway to the Amazon’, Belém was a strategically symbolic venue for COP30, expected to spotlight the needs of the Amazon, remind the world of the value of rainforests and highlight the Brazilian government’s own efforts at protecting the forest [43]. It was hoped by Brazilian authorities that hosting the COP in Belém would fortify the climate–nature connection and accelerate progress on tackling global deforestation [43]. Nevertheless, the resolve to use cruise liners to plug the accommodation gap in Belém highlighted the tensions between COP’s stated vision of being a ‘People’s Conference’ and the reality that the logistical issues in Belém were likely to restrict access to some people and result in unequal participation.

Of 3900 cabins on both cruise liners, 15 cabins were reportedly allocated to each Least Developed Country (LDCs) and Small Island States (priced at US$100–220 per night), with ten for other delegations at US$230–600 per night [44]. Moreover, due to US sanctions on 20 countries – including North Korea, Cuba, Iran, Haiti, Chad, Somalia, Sudan, etc. – a number of delegations were prohibited from booking housing on the *Costa Diadema* [45]. The exclusion is linked to the ship ownership and operators, who are financially and logistically linked to the US, and hence are required to comply with the US Office of Asset Control (OFAC) sanctions regime [45]. According to OFAC rules, entities linked to the US are prohibited from trading with governments or individuals under general or specific sanctions. However, there were no such bans associated with the *MSC Seaview* due to its comprehensive European ownership and operation. Such inconsistencies only served to intensify criticisms that housing in COP30 was uneven and exclusionary.

Indeed, civil society organisations (CSOs) and negotiators from developing countries cautioned that there was a risk of the climate summit becoming an ‘exclusive COP’ – only accessible to participants with large budgets or special diplomatic status. After weeks of uncertainty, one African negotiator lamented that the planning for COP30 seemed to only consider the media and CSOs as an afterthought [8]. Equally, an international policy coordinator at Observatorio do Clima (a CSO climate coalition) described the logistical unpreparedness of Brazilian authorities for COP30 as ‘the antithesis of a people’s COP, an inclusive COP’, concluding that ‘this COP risks being the most exclusionary in the history of the UN climate convention’ [46]. Compounding these challenges were the extraordinary surge in prices of basic apartments in Belém, with fees for two weeks costing over US$250,000. Responding to this issue, a collection of 25 countries led by the African group made an official request for the relocation of the conference, making the case that financial and logistical barriers would compromise equitable participation [45]. While Brazilian authorities rejected this request, they offered partial subsidies that many critics still found to be inadequate [8].

This issue was also partly reflected in a letter from the Alliance of Small Island States (AOSIS) to the presidency of COP30, detailing their worries about ‘affordability and equitable participation’ due to the high prices of hotel rooms, and outlining that delegates from AOSIS were unsure of their ability to attend, and that SIDS delegates were struggling to secure appropriate accommodation [47]. Indeed, official documentation from the UNFCCC in the aftermath of the conference shows a large registration-to-attendance drop associated with last-minute attrition driven by costs and logistics concerns: UNFCCC provisional on-site participation registrations totalled 56,118, but the final conference in-person attendance totalled 42,618 [48,49]. Official data from the IBGE also shows that Belém’s regional inflation spike was driven by accommodation (+155.24%) over the course of the COP period [24].

Furthermore, as Campos et al. [50] aver, there is a symbolic paradox in Brazil’s use of cruise liners in Belém. In their analysis, they echo similar sentiments about emissions from travelling to COPs [6,7,15], arguing that the plan was in sharp conflict with the ‘spirit of global climate targets’. Collins et al. [51] highlight the tensions between the symbolic goals of global conferences such as COPs and the environmental impacts generated by travelling, infrastructure and logistics associated with such events. Indeed, the analysis of cruise ships above reiterates their arguments: the operation of cruise ships generates more carbon per passenger than most means of transportation. Some observers are worried that much like the private jet controversies of COP26 and COP27 (see [15]), the use of cruise ships reflected ‘climate elitism, or even COP as an exotic holiday – floating five-star hotels’ for diplomats at a summit dedicated to justice and sustainability.

Furthermore, having COP30 in Brazil was publicised as a necessary counter to Global North dominance in climate negotiations, a symbolic inclusion of countries at the frontline of climate struggles. Paradoxically, however, electing to host the summit in Belém (remote and limited in logistical capacity), seems to have eroded the very inclusivity it hoped to foster. While COP26 in Glasgow and COP27 in Sharm el-Sheikh were heavily criticised for travel [7] and civic-space barriers [52], the challenge for COP30 went a step further in terms of material access to include both accommodation and affordability. As Bompan [53] writes in an op-ed for Renewable Matter, a combination of distance and high costs were likely to drastically cut attendance to COP30, especially from grassroots groups and independent media. While CSOs utilised Belém’s public spaces to host an independent ‘People’s Summit’ during the conference [54], such a segregation was always unlikely to have any influence on formal negotiations.

The logistical challenges faced by COP30 and the controversy over the use of cruise liners to house delegates once more highlighted the chronic tension between symbolic inclusion and structural exclusion in climate negotiations and global governance. When the capacity to participate is almost wholly dependent on financial means rather than representation, the process risks becoming delegitimised. As critics have observed, the pricing out of many potential participants outside international delegates will likely shift the ‘balance of voices’ to the benefit of those with strictly national interests that prefer weaker climate action [46]. Such a dynamic will breed mistrust as well as potentially skew negotiations to the benefit of better resourced parties. The material constraints associated with hosting the conference in Belém raised long-standing concerns about just and equitable participation in global climate governance. Scholarship shows that inequalities in capacity and representation informs who gets to participate and how states are represented in environmental negotiations [3,55]. Broadly, research on post-Paris ‘hybrid’ climate governance shows that participation structures can both enable and constrain actors, raising questions of justice and legitimacy [4,55]. In this regard, COP30 is illustrative of how the material set-up of climate diplomacy – from travelling logistics to housing infrastructure – can shape both emissions outcomes and the inclusivity of participation in climate negotiations.

### Contextualising COP30 in terms of travel and accommodation

The remote location of COP30 posed similar challenges encountered in previous UN climate summits in terms of travel; but also presented a further and unique complication in terms of accommodation. Barnsley et al. [7] showed that COP26 in Glasgow, up to that time, had represented the largest carbon footprint from any COPs, averaging around 3.42 t CO_2_e per participant. The high figure as noted in their analysis reflected enhanced and more transparent reporting – COP26 was the first to fully attempt to account for all emissions from international travel (utilising a radiative forcing factor for flights) in calculations, generating 150% more footprint than COP25, and with 75% of emissions coming from travel by international delegates. In contrast, COP27 in Sharm El-Sheikh yielded substantial emissions from travel under different contexts. The conference location on the Sinai Peninsula meant that for most delegates flying was inevitable; while conflict in bordering nations and a lack of ferry connections also meant on the ground low-carbon transportation was practically impossible [7]. This is in sharp contrast with Glasgow, where delegates from Europe could in reality travel by coach or rail: taking the train from London, for example, cut emissions by up to 64% [7]. Such options were unavailable to delegates travelling to COP27 and goes to demonstrate how conference venues and logistical infrastructure have a direct impact on carbon footprint per participant.

Significantly, previous COPs and especially COP30 in Belém highlight how the choice of host city for summits can influence who can attend, as well as how they might be accommodated. COP26 in Glasgow recorded high levels of participation at that time (approximately 40,000) but faced criticism around equity issues. As a high-income country, travel and accommodation costs in the UK posed a challenge to participants from the Global South [7]. While a stopgap such as the establishment of a ‘Human Hotel’ homestay network provided affordable housing for 1696 participants who otherwise would have been priced out of attendance [7], it underlines the necessity of taking deliberate measures to mitigate accessibility challenges and to ensure that participants from less well-off contexts are not marginalised by prohibitive costs. Similar challenges recurred in Sharm El-Sheikh, whose status as a tourist destination ensured accommodation availability, but at an exorbitant price. In response, the Egyptian government in coordination with the UN instituted a price cap on hotel rates [56]. In Dubai, however, COP28 enjoyed the United Arab Emirates’s extensive accommodation capacity; nonetheless, the unprecedented number of participants (84,000) meant more long-haul flights along with a rise in the use of private jets [6]. Five hundred and eighty private jets are reported to have flown in and out of Dubai for COP28, yielding an estimated 14,000 t CO_2_e (14 kt) over the course of the conference [6]. COP28 rightly drew criticism for undercutting the conference’s message on climate change, as well as highlighting the inequality in VIP travel.

Additionally, from COP29 in Baku (2024) and now to COP30 in Belém (2025), conference organisers have had to grapple with infrastructural constraints and rein in participant numbers. While Baku is in Eastern Europe, direct flights from the UK are limited to one airport (London Heathrow) and one airline serviced route (Azerbaijan Airlines); while also across Europe only a limited number of direct flights to Azerbaijan were available [6]. The implications for participants from the UK attending COP29 was that their individual average emissions increased compared to travelling to Dubai for COP28 (Dubai is a global aviation hub with multiple daily flights from around the globe), even though Baku is 1500 km closer to London [6]. For Baku, nonetheless, participants had options of cutting emissions through a combination of train and ferry for example, albeit with certain costs such as affordability.

Previous research have engaged with the scale, necessity and political economy of COP summits, raising broader questions about the organisation of climate diplomacy. Participation at COPs has expanded well beyond core negotiators, encompassing a wider range of actors. As some studies note, a broadened participation has the capacity to enhance legitimacy but at the risk of potentially raising issues of equity and capacity [3,55]. Research on international conferences and mega-events consistently demonstrates that travel typically represents the dominant source of associated emissions [57,58]; in this context, travel and accommodation for COP30 illustrates some of these tensions – legitimacy, inclusivity and sustainability. This has led to calls for exploring new models of hosting COPs: hybrid participation models, regional negotiation hubs or scaled-down in-person delegations, to help cut emissions while maintaining substantial participation in global climate diplomacy [59–61].

Moreover, in anticipation for COP29 and COP30, the UNFCCC and host governments opted to put a cap on numbers to between 40,000 and 50,000, with accreditation cuts to observer groups, and a purposive reallocation of badges from Global North non-governmental organisations to less represented groups from the Global South [6]. The move sought to both foster principles of equity, and ease infrastructural pressures. As Brown et al. [6] posit, the decision moves the climate conference process closer to principles of climate justice; yet COP30’s provision of two cruise ships (3900 cabins/6000 beds), and prioritisation of the LDCs and Small Island States did not do much to encourage and make participation easier for many from the Global South. The floating hotels were only available to international delegates, and many negotiators from vulnerable countries lamented that the $US150–220 per night cap was still significantly beyond their budgets [8]. Moreover, the ships were moored 20 km away from the conference venue, requiring regular road trips on congested roads [45]. The UNFCCC’s shift in emphasis on climate justice in terms of representation in negotiations is admirable; however, as COP30 in Belém demonstrates, poor logistical planning can often exacerbate the problem.

## Recommendations and conclusion

The 2025 annual UNFCCC climate summit (COP30) in Belém highlights the persistent paradox that underlines global efforts against climate change, in a carbon-intensive and unequal world. Setting COP30 in Belém represented at least a symbolic triumph – an opportunity to spotlight the Global South and the most climate-vulnerable regions. Yet the logistical practicalities of hosting it there – long-haul flights, inadequate housing and the controversy of utilising cruise ships as a housing solution – potentially undermined that aspiration. Research has long shown that large international events generate substantial environmental impacts, especially through travel and temporary infrastructure [51]. Moreover, studies on event sustainability also highlight the challenges associated with cutting environmental impacts where large numbers of participants have to travel long distances [62]. As Nevins [57] has previously shown, travel often dominates emissions for international conferences; and this paper, just as previous ones in this series – Glasgow [7], Dubai [15] and Baku [6] – demonstrates this claim using COP30 in Belém. The paper also shows that the carbon footprint and accessibility of every conference is influenced by the geography, infrastructure and governance choices of the hosts. In the case of Belém, remoteness did not only generate high emissions from travel, but improvised housing solutions also magnified the emissions.

Indeed, travel will remain the single largest source of emissions across COPs (70–80% of cumulative footprints), but Belém adds a significant dimension in terms of accommodation-related emissions from the *MSC Seaview* and *Costa Diadema*. Both cruise liners are expected to have generated hundreds of tonnes of CO_2_e even while moored. This is in line with findings from Collins et al. [51], who note that temporary infrastructures at conferences tend to contribute significantly to emissions. This is also in line with concerns over infrastructure and high-carbon lock-in: according to Seto et al. [63], investments in infrastructure associated with mega-events can contribute to long-term ‘carbon lock-in’ by reinforcing high-emission systems and practices. Hence, the logistical measures necessary to host COP30 – including using the cruise ships for temporary accommodation – illuminates how major climate summits may reproduce carbon-intensive infrastructure even as they go about trying to address climate change. This study has used established analytical tools to interrogate the ways that logistical arrangements around COP30 elucidate broader structural issues in contemporary climate governance, especially as it pertains to harmonising participation in mega summits with the environmental impacts of global climate negotiations.

Nonetheless, Belém offers four valuable lessons for the UNFCCC, especially if it is to stay true to its principles of equitable representation.

First, host locations should be able to balance symbolic inclusion with sustainability connectivity. As previous studies in this series show, locations accessible by low-carbon routes (rail or coach), tend to significantly cut emissions [6,7,15]. Previous research on international meetings and mega-events also reveal that a host city can substantially impact aggregate emissions associated with travel distances [51]. In principle, the selection of the COP host could consider integrating geographical optimisation, such as selecting a location that reduces aggregate distances or utilising a rotating system that distributes travel burdens more evenly among participants. The current UNFCCC system operates an informal regional rotation system for COP presidencies, but the material realities implies that travel distances still significantly vary across host locations. As highlighted in some studies, a regional rotation model whereby international mega conferences alternate between global regions can bridge the goals of inclusivity and accessibility while cutting the aggregate travel burden placed on specific regions over time [4]. The UNFCCC could introduce some standard sustainability requirements, such as low-carbon regional hubs or request that each region have at least one location that can be accessed by electrified rail networks.

Second, Belém shows that, in certain contexts, accommodation can substantially expand the impact of emissions from travel. Brown et al. [6] have previously underlined this as a methodological problem: carbon footprint calculators in the future must also account for ‘stationary energy consumption’ at conferences. These would include in-situ energy utilisation in hotels, conference centres and temporary facilities. Such a move towards clear and standardised carbon accounting would strengthen the credibility of UN climate summits.

Third, as recommended in previous papers in this series, providing hybrid and digital participation as a mainstay in COPs can substantially reduce emissions and simultaneously widen accessibility to marginalised groups. Over time, participation in COPs has expanded to include CSOs, businesses and media representatives. This expansion highlights general trends towards multi-actor climate governance, but at the risk expanding the logistical complexities and environmental footprint of climate diplomacy [3,4,64]. While virtual or digital participation are not without their own sources of emissions, evidence from conferences that moved online during the Covid-19 pandemic demonstrates that eliminating travel significantly cuts the carbon footprint of mega-events [65], while expanding participation among geographically distant or resource-constrained participants [66]. Moreover, a hybrid system that connects regional multi-hub locations digitally has been evidenced to cut travel-related emissions by around two-thirds in comparison to single-location conferences [67].

And fourth, in the same spirit as previous papers in this series, it is requisite that conference organisers commit to transparent, standardised carbon accounting, comprising verified offsets for sustainable co-benefits. Each host should produce a forecast report detailing the potential emissions they foresee the conference generating, and within one year of the conference be required to publish an independently verified carbon footprint report. Both reports can provide significant information that can be fed into preparations for future COPs, especially in terms of lessons learned. Such a move would also improve transparency and allow for progress tracking over time.

This study is not without some limitation or the other: two are worth highlighting. First, the analysis of emissions from cruise ship accommodation is wholly derived from estimates of hotel-load energy use in the extant literature, rather than specific vessels’ operational data. Therefore, the values examined here should be interpreted primarily as indicative approximations rather than precise measurements. Access to empirical operational data on auxiliary engine use or port electricity consumption would help future studies to refine these estimates. Second, one of the study’s main focuses is emissions associated with travel to COP30 from UK origins only. Hence, the study does not account for a more global and larger travel footprint to the summit in Belém. Participants from other regions of the world are expected to experience completely different routing patterns as well as associated emissions. Future studies could extend this approach by modelling travel from an extended range of geographical origins so as to better account for the global aggregate distribution of emissions associated with conference participation.

Crucially, future studies must continue to explore alternative organisational models for global climate negotiations, including the practicalities, associated risks and potential emissions from digital (hybrid) participation, or scaled-down in-person (multi-hub digital regional models) delegations expanded by larger digital participation. A comparative examination of various models could be useful for assessing the extent to which hybrid or scaled-down formats might help cut travel and accommodation-related emissions, while ensuring the efficacy and inclusivity of climate negotiations. Finally, the incongruities that underlined COP30 in Belém reflect those common in global climate governance: processes that seek to promote sustainability, tend to be hindered by the systems they seek to transform. Nonetheless, cutting emissions from COPs is not merely symbolic, it is the sine qua non without which all efforts at climate diplomacy collapse.

## Data Availability

The datasets generated during and/or analysed during the current study are available from the corresponding author on reasonable request.

## Appendix

### Emissions calculation methods and assumptions

#### Key assumptions

- Unit of analysis: Per-delegate emissions for a round-trip journey from the UK (baseline: London) to Belém and accommodation during the COP30 period [7].
- Aviation dominates travel emissions: Emissions are estimated per-aircraft by summing LTO cycle fuel burn plus cruise fuel (using the ICAO method). We assume economy-class seating for all delegates (premium cabins not modelled) and use published emission factors to convert fuel burn to CO_2_ per passenger [31].
- Routing: London–Belém routes are modelled via commercially plausible hubs (e.g., Lisbon; Brazilian domestic hubs), based on flight availability on major flight network trackers (FlightConnections, Google Flights, FlightRadar24).
- Non-CO_2_ radiative forcing is excluded from the core totals: Department for Environment, Food and Rural Affairs’ (DEFRA) 1.7× multiplier is shown only for comparison, while Barnsley et al.’s [7] suggested ×2 ‘climate multiplier’ is noted in sensitivity discussions.
- Hotels accommodation emissions are estimated using DEFRA/DESNZ’s per-night ‘hotel stay’ factors and standard occupancy assumptions (one delegate per room). We apply factors of 10 kg CO_2_ per occupied room-night (typical for 3–4 star hotels) based on UK guidance.
- Cruise ships: Cruise accommodation is treated as scenario-based hoteling emissions (at-berth operation) bounded using literature and fuel emission factors; voyage/sailing emissions are excluded [26].
- Overland travel in Europe is omitted as negligible relative to intercontinental flights.
- Contextual evidence (not emissions inputs): UNFCCC participation documents are used to report registered vs. actual participation; IBGE inflation releases are used to document COP-period price shocks in Belém (see Table A1).

**Table A1.** UNFCCC provisional and final in-person attendance numbers for COP30.

Delegate category	Provisional registered	Final in-person
Party delegations (national)	30,200	28,500
Observer organisations (NGOs, IGOs)	18,700	15,800
Media and others	3100	2400
Total in-person	52,000	46,700
Virtual (online)	22,500	15,000

*Source:* UNFCCC COP30 Secretariat (provisional and final attendance reports).

### Emissions accounting boundary and reporting conventions

All emissions are reported in kg CO_2_ e (and where relevant, also referenced as CO_2_-only components). Aviation and hotel factors are drawn from the UK Government’s annual conversion factors for company reporting (DESNZ/DEFRA), which provide standardised factors for direct emissions and, where available, well-to-tank (WTT) additions.

The analysis distinguishes between: quantified aviation emissions estimates for specified UK–Belém routes (calculator-based, using standardised factors); and literature-based scenario analysis for cruise ship accommodation emissions, reflecting limited vessel-specific operational data availability.

### Aviation emissions calculation

#### Emission factors used for flights

The primary flight emission factors are taken from the UK Government GHG Conversion Factors (2024) ‘Business travel – air’ tables (Scope 3), reported per passenger-km and disaggregated by seating class and RF treatment.

- Baseline factors applied (direct, without RF uplift):
  - Short-haul, economy: 0.10794 kg CO_2_ e/pkm
  - Long-haul, economy: 0.11812 kg CO_2_ e/pkm
- Sensitivity (direct, without RF uplift):
  - Long-haul, business: 0.34252 kg CO_2_ e/pkm
  - Long-haul, first: 0.47246 kg CO_2_ e/pkm

Where WTT emissions are included in sensitivity calculations, DEFRA’s ‘WTT – business travel – air’ factors were added (e.g., long-haul economy WTT = 0.02461 kg CO_2_ e/pkm).

#### Distance and routing uplift

Distances between airports are computed as great circle distance (GCD). Following DEFRA methodology guidance, an 8% uplift is applied to account for indirect routing, delays and circling (i.e., distance_effective = GCD × 1.08).

#### Core calculation expression

For each flight segment *i*:

$$E_{i}=({\mathrm{GCD}}_{i}\;\times \;1.08)\;\times \;EF_{i}$$

where *EF_i_* is the relevant DEFRA factor (kg CO_2_ e/pkm) for haul length and class (and RF choice).

Route totals are calculated by summing segments and multiplying by 2 for round-trip estimates.

#### Flight class and load factor assumptions

This paper uses economy-class factors as the baseline because economy seating density is the most conservative standard allocation for representative delegates and aligns with the calculator approach used in earlier papers in this series (default London start point; adaptable network model).

Where the ICAO approach is referenced for methodological alignment, ICAO’s calculator methodology allocates fuel to passengers using load factors and passenger-to-cargo factors derived from ICAO operational data and converts fuel to CO_2_ using 3.16 (kg CO_2_ per kg fuel) [21].

#### Radiative forcing treatment for aviation

- Base case: Aviation emissions are reported using DEFRA conversion factors without radiative forcing uplift. These factors include combustion GHGs (CO_2_, CH_4_ and N_2_O) expressed as CO_2_ e but exclude wider non-CO_2_ aviation effects such as contrails and aviation-induced cirrus.
- Sensitivity case: To illustrate the potential magnitude of non-CO_2_ aviation impacts, DEFRA’s radiative forcing approach is applied either by (a) using the DEFRA ‘With RF’ flight emission factors, or (b) applying the indicative 1.7× multiplier provided in DEFRA guidance to approximate additional aviation climate effects beyond combustion GHGs.

This sensitivity is reported as an uncertainty range because DEFRA explicitly notes significant scientific uncertainty in the magnitude of non-CO_2_ effects, and the IPCC AR6 identifies contrails and aviation-induced cirrus as a distinct forcing agent with uncertainty bounds.

#### Land-based hotel accommodation emissions

Hotel emissions are estimated using DEFRA/DESNZ ‘Hotel stay’ factors (Scope 3), reported in kg CO_2_ e per room-night (country-specific average).

For accommodation in Brazil, the analysis uses the UK Government GHG conversion factor for international hotel stays.

The paper assumes: one room per delegate (single occupancy), *N* (nights) corresponding to the relevant stay duration. Thus:

$$E_{\text{hotel}}\;=\;8.7\;\times \;N\;({\text{kg CO}}_{\text{2}}\text{e})$$

#### Cruise ship accommodation: scenario-based hoteling emissions

Because ship-specific fuel logs, auxiliary power loads and shore-power usage during COP30 were not publicly available to the authors, cruise emissions are presented as bounded scenarios, not definitive totals.

#### Boundary: hoteling versus sailing

The cruise ship component is defined as at-berth hoteling emissions associated with using cruise ships as floating hotels (powering lighting, HVAC, services).

Voyage/sailing emissions (including repositioning to Belém) are excluded unless explicitly modelled, because the ships’ prior itineraries and exact repositioning routes are uncertain. This boundary responds directly to methodological concerns about mixing sailing and in-port emissions.

Industry studies indicate that large cruise vessels maintain significant auxiliary engine loads while docked in order to power onboard hotel services [26]. ICCT’s travel analysis (e.g., 317 g CO_2_/pax-nm) and Friends of the Earth’s (FOE) reports (421 kg CO_2_ per passenger-day) typically combine propulsion and hotel energy demand during voyages, rather than one or the other only.

#### Fuel and onboard generation assumption

COP30 reporting indicates the ships relied on onboard generators for onboard services, with fuels ‘ranging from conventional diesel to biodiesel’ [8].

#### Scenario boundaries

- Occupancy basis: COP30 Secretariat materials indicate that approximately 6000 accommodation places were allocated across two cruise ships, equivalent to roughly 3000 guests per vessel for modelling purposes [44].
- Hoteling fuel burn bound (large ships at berth): Simonsen et al. [26] discuss at-berth auxiliary fuel consumption for large cruise ships and note that estimates differ across surveys; for large ships, reported at-berth fuel consumption ranges on the order of 1.5–1.9 tons fuel per hour depending on underlying assumptions and datasets.
- Fuel-to-emissions factor: The UK Government fuel factors provide diesel (average biofuel blend) as 3204 kg CO_2_ e per ton (direct), used here as an approximation for diesel/biodiesel combustion in onboard generation [31].

Per-ship daily hoteling emissions (bounded):

$$E_{\text{ship, day}}\approx (1.5\text{ to }1.9)\;\frac{\text{t}\;\mathrm{fuel}}{h}\;\times \;24\;\text{h }\times \;3.204\;\frac{\text{t}\;{\text{CO}}_{\text{2}}\text{e}}{\text{t}\;\mathrm{fuel}}$$

115–146 t CO_2_ e per ship-day (at berth) following Simonsen et al. [26].

Per-guest-night hoteling emissions (bounded):

$$E_{\text{guest, night}}\approx \frac{E_{\text{ship, day}}}{3,000}$$

38–49 kg CO_2_ e per guest-night (per ship; assumes full occupancy).

These values are presented as indicative scenario bounds rather than precise operational measurements. For context, broader cruise-vacation analyses – such as FOE’s estimate of 421 kg CO_2_ per passenger-day – include both propulsion and hotel energy demand and therefore represent a substantially wider system boundary than the hoteling-only estimates presented here (see [9]).

#### Treatment of excluded components and key limitations

- European overland travel exclusion: Ground transport within Europe is excluded because the analysis focuses on the dominant long-haul aviation component of travel emissions, consistent with the modelling approach used in earlier papers in this series. For intercontinental journeys such as UK–Belém, aviation emissions exceed surface transport emissions by orders of magnitude, meaning that inclusion of short European ground segments would not materially alter the comparative results.
- Key limitations and uncertainty quantification:
  - Aviation: Results are sensitive to (a) passenger booking class (economy vs. premium), (b) route choice and associated detours, approximated using an 8% distance uplift to reflect real-world routing, and (c) treatment of radiative forcing, which is reported as a sensitivity case rather than a single definitive value.
  - Hotels: Hotel factors are country-level averages and do not capture specific property performance; single vs double occupancy materially changes per-person allocations.
  - Cruise ships: Hoteling estimates are bounded but still uncertain due to unknown auxiliary load profiles, actual occupancy, fuel blend, and any shore-power availability/uptake. Voyage/repositioning emissions are omitted unless explicitly modelled (to avoid conflating sailing and in-port emissions).
  - Comparability: The calculator-based approach is designed for transparency and comparability, not ship/operator-grade auditing; therefore, cruise outputs are presented as scenario ranges and interpreted with caution.

#### Calculator links

- https://www.ucl.ac.uk/climate-change/partnerships/ucl-cop
- https://www.ucl.ac.uk/climate-change/partnerships/ucl-cop/cop30-carbon-footprint-calculator

#### Sources

- UK Government (DESNZ/DEFRA). *Greenhouse gas reporting: conversion factors 2024* (incl. flat-format spreadsheet and methodology). https://www.gov.uk/government/publications/greenhouse-gas-reporting-conversion-factors-2024
- Flat-format factors file (used for numeric factors): https://assets.publishing.service.gov.uk/media/6722567c10b0d582ee8c4953/ghg-conversion-factors-2024-FlatFormat_v1_1.xlsx
- Methodology paper: https://assets.publishing.service.gov.uk/media/66a9fe4ca3c2a28abb50da4a/2024-greenhouse-gas-conversion-factors-methodology.pdf
- ICAO. *ICAO Carbon Emissions Calculator (ICEC) and Methodology v13* (fuel-to-CO_2_ conversion and passenger allocation principles). https://www.icao.int/environmental-protection/environmental-tools/icec
- Methodology PDF: https://icec.icao.int/Documents/Methodology%20ICAO%20Carbon%20Emissions%20Calculator_v13_Final.pdf
- UNFCCC. *COP30.PLOP Provisional list of registered participants* (10 Nov 2025). https://unfccc.int/sites/default/files/resource/PLOP_COP30.pdf
- UNFCCC. *FCCC/CP/2025/INF.1 List of participants* (9 Dec 2025). https://unfccc.int/sites/default/files/resource/cp2025_inf01.pdf
- COP30 Secretariat. *Cruise ships arrive in Belém to boost accommodations* (5 Nov 2025). https://cop30.br/en/news-about-cop30/cruise-ships-arrive-in-belem-to-boost-accommodations-for-cop30
- Friends of the Earth. *Cruising Versus Land Vacationing* (2023) (comparative per-day cruise vs land footprints). https://foe.org/wp-content/uploads/2023/04/Comparison_of_CO2_Emissions_v2-1.pdf
- Simonsen, M. et al. *Model for Estimation of Fuel Consumption of Cruise Ships* (Energies, 2018) (in-port fuel consumption modeling). https://www.mdpi.com/1996-1073/11/5/1059
- Climate Home News. *Brazil offers COP30 cruise ship rooms and cost caps…* (18 Jul 2025) (onboard generators; fuel range claim attributed to COP30 spokesperson). https://www.climatechangenews.com/2025/07/18/brazil-offers-cop30-cruise-ship-rooms-and-cost-caps-but-negotiators-remain-unhappy/
